# Team-based learning-adopted strategy in pharmacy education: pharmacology and medicinal chemistry students’ perceptions

**DOI:** 10.1186/s43094-023-00464-6

**Published:** 2023-02-23

**Authors:** Reem T. Attia, Asmaa A. Mandour

**Affiliations:** 1 Pharmacology and Toxicology and Biochemistry Department, Faculty of Pharmacy, Future University in Egypt (FUE), Cairo, 11835 Egypt; 2 Pharmaceutical Chemistry Department, Faculty of Pharmacy, Future University in Egypt (FUE), Cairo, 11835 Egypt

**Keywords:** Team-based learning, Pharmacology, Medicinal chemistry, Readiness assessment test, Interactive learning

## Abstract

**Background:**

Team-based learning (TBL) provides an advanced teaching method for healthcare education; it is characterised by being an interactive teaching session that allows groups of learners to work together in teams to discuss and apply what they have learnt to certain clinical scenarios. The following study aims to evaluate the impact of TBL strategy on the students' comprehension and acquired knowledge, to allow better application and integration of knowledge. The aim of the study was to improve pharmacy students' skills in achieving learning outcomes by adapting TBL pedagogy in the lectures. Students’ feedbacks were collected via post-lecture survey.

**Results:**

The study was applied to pharmacy students covering two courses: Pharmacology III (Level 4) and Medicinal Chemistry I (Level 3) in a period of two-week lectures through the first semester of the academic year 2021/2022 in Future University in Egypt. The selected topics-related preparation materials were previously available on each course moodle page prior to the actual lecture, for the students to get prepared including growth hormone, sex hormones and their associated diseases for the pharmacology course and COVID-19 management for the Medicinal Chemistry course. The TBL lecture was started by dividing the students into teams and then readiness assurance tests were given, as individual readiness assurance test and then team readiness assurance test conceptual test were applied. The assessment of the students’ decision-making skills and problem solving was evaluated through solving-related clinical cases. All the learning outcomes were achieved with maximum participation and interaction via an open discussion between the lecturer and the students during the lecture. A total of 116 students answered the survey and confirmed their satisfaction, better understanding and more participation in TBL lectures compared to other topics taught with the ordinary methods. More than half of the students recommended the TBL method for better perception and participation.

**Conclusion:**

The students felt great appreciation for the team-based lecturing. Also, recommendations and suggestions were directed towards increasing the percentage of TBL lectures in the curriculum, as it helped them to concentrate more with high participation levels.

## Background

The recent technological transformations in the society had significant impacts on students’ demands and expectations. Teaching–learning relationship required huge changes to upgrade student profile and to keep the students focusing during lectures [1, 2]. Medical education has adopted new and active methodologies other than traditional teaching ways. The aim of these practises is to improve learning by keeping students engaged through different ways [1, 2]. Active-learning approaches target higher cognitive level knowledge, besides skills development [3, 4]. By allowing students to formulate questions and to test them in open conversations rather than the one-way lecture. Team-based learning (TBL) is a methodology that has been implemented in many medical and health schools, including medicine, dentistry, pharmacy and nursing [5–8].

TBL encourages application and integration of knowledge through student’s group work in a single session [9], where groups of students could be divided into small teams. Then questions in different forms could be directed first to students individually, then to teams, and finally the groups in open discussions can cooperate in solving the applied problems [1, 2].

It is believed that instructors should find ways for meaningful active learning approaches in their classes. Active learning strategies should take into consideration course objectives, teaching styles, and students' level of experience [10].

The main outcome here is that our efforts were targeted to the medical students including pharmacy students, who need active, student-centred curriculum that can attract students’ interest and engagement in learning. Hence, this results in better short- and long-term learning outcomes. Based on the fact that the practised active learning approaches for years, all efforts should be continued in order to modify passive teaching to new, active, student-centred strategies. It’s worth noting that many studies and researches confirmed students’ enthusiasm for this curriculum reform [10].

## Methods

The presented study aimed to evaluate the difference in students’ satisfaction, understanding and participation in lectures applying TBL during two weeks of the semester. Here two different topics of Pharmacology III course: growth hormone, sex hormones and their associated diseases, were taught to fourth level students at the Faculty of Pharmacy, Future University in Egypt. Also, a Medicinal Chemistry I topic named COVID-19 Management was taught to the third level students at the same campus chosen to conduct TBL through the first semester of the academic year 2021/2022.

Each instructor previously educated his students about the TBL method of learning, one week prior to the aimed lecture timing. The concept was put to action through adding some topic-related readings; the theoretical lecture pdf together with topic-related links, videos and other preparation materials were available on the course moodle page before the actual lecture as well. This allowed the students to get prepared before the lecture and be aware of most of the needed knowledge about the targeted topics. The instructor then started the lecture by distributing students among different teams and naming the different teams who will work together through the whole lecture. After that, the students took the readiness assurance tests (RAT), starting by an individual (iRAT) and then a team (tRAT) conceptual test in order to assess their understanding of the pre-class material.

The questions covered the specified topic and included a clinical case that assessed the comprehension of the students to the studied topic. The students were given the time to discuss the answers to the given case with their groups in the same time.

Student groups were encouraged to co-operate to problem solve and justify their answers with effective interpretation to the supplied pre-class material.

Points were given for all students, and the team with the highest points was selected as the winner team to encourage the students’ enthusiasm and eagerness to learn. Finally, the questions were answered in an open discussion between the lecturer and the students and each team was given full 5 points on each correct answer from the first trial. If the answer was incorrect, the team then discussed the other answers and they get only 4 points for the correct answer and so on. Thus, the number of points is inversely proportionate to the number of trials. Through the questions and answers session, the whole outline of the lecture was discussed, and the learning outcomes were fulfilled.

**Fig. 1:**
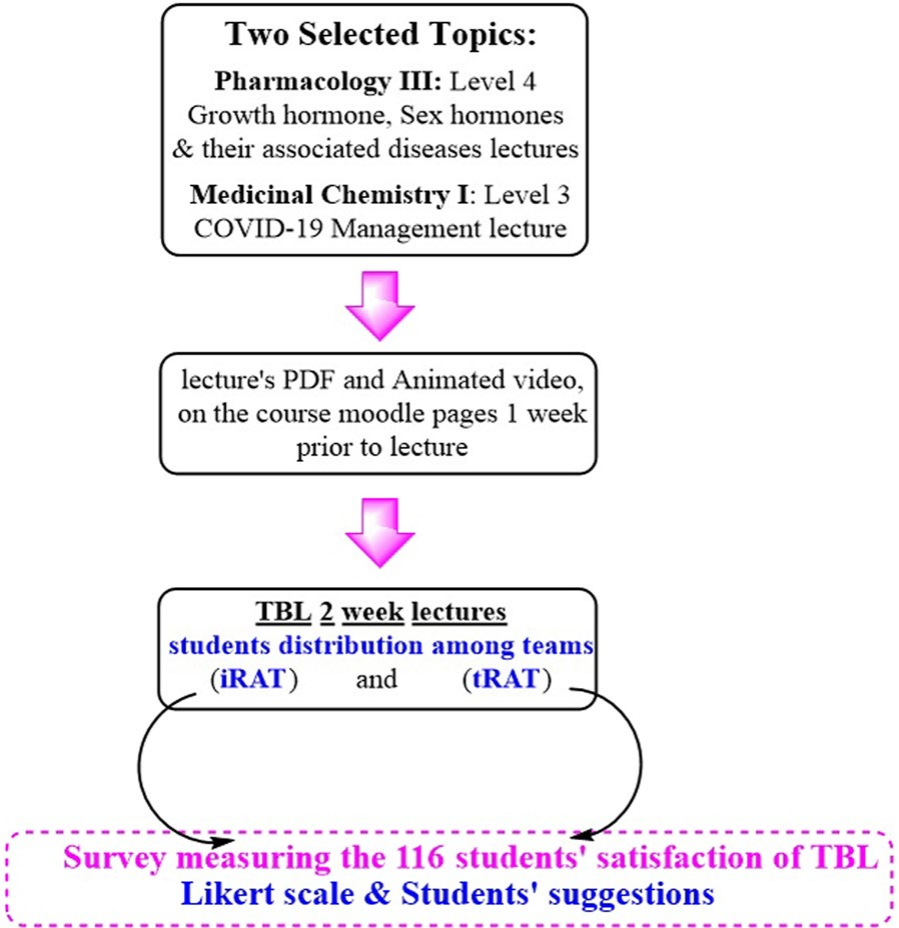
A schematic representation of methodology design for TBL lectures. iRAT: Individual Readiness Assessment Test, tRAT; Team Readiness Assessment Test, TBL: team-based learning

Finally, a survey was collected measuring the students’ satisfaction about the concept of TBL. The results were spectacular, showing full understanding and participation from the students’ end (Fig. 1).

### Data acquisition

Post survey results were collected from 116 students after their session of TBL in both courses. The post survey consisted of 11 points Likert scale questions and one open-ended question; these questions were assessing, communication among teams, how using TBL influenced their understanding, the fulfilment of learning outcomes and overall satisfaction among students.

### Statistical analysis

The statistical analysis was done using Microsoft Excel and GraphPad prism software. The data are presented as means ± SD.

## Results

The experiences of 116 students were collected in a survey during the team-based learning lecture. The students’ responses were classified according to their assessment concerning the team-based session that they attended during their courses.

**Fig. 2:**
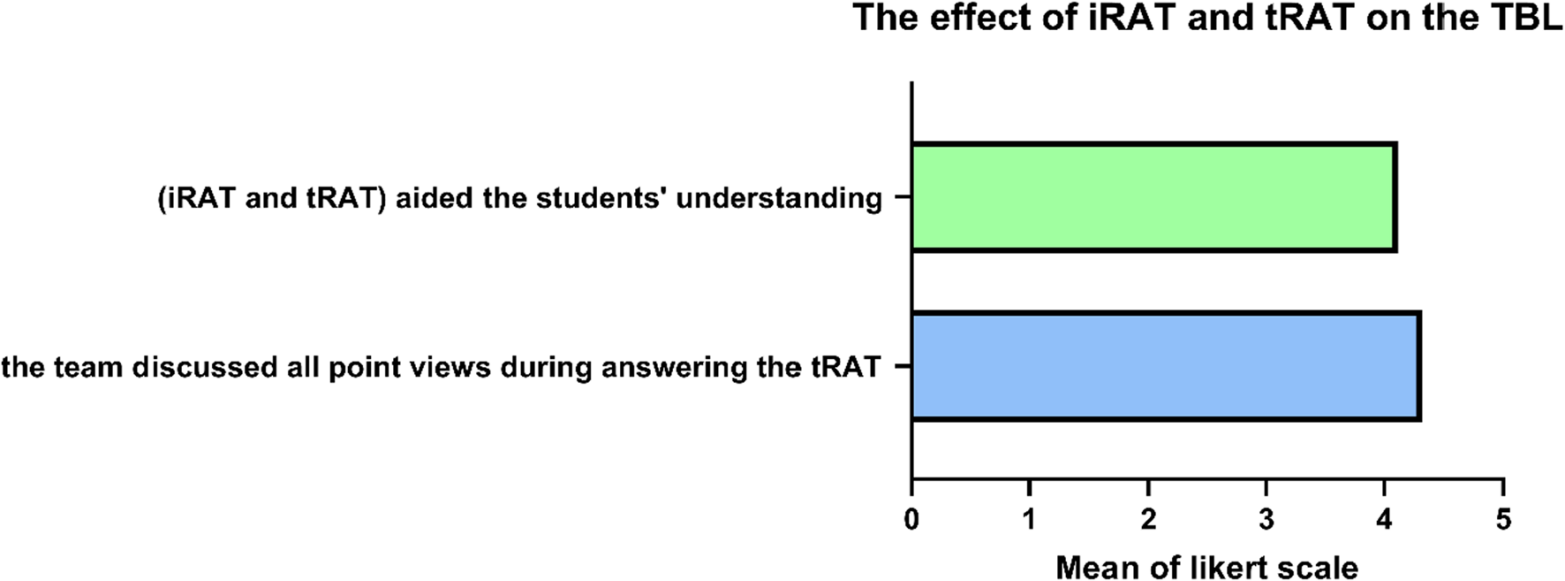
The effect of iRAT and tRAT on TBL. Data are presented as mean ± SD. *n* = 116. iRAT: Individual Readiness Assessment Test, tRAT: Team Readiness Assessment Test, TBL: team-based learning, where 1 is the least and 5 is the most

First of all, the effect of both readiness tests, the iRAT and the tRAT, was measured according to the students; around 88% of the students either strongly agreed or agreed that those tests were important tools for their understanding of the topic, and 86.2% of the students either strongly agreed or agreed that the team discussed all point of views during answering the tRAT tests (Fig. 2, Table 1).

**Table 1:** Post-lecture survey for TBL

Type of question	Contents	Value	Median	Mode
Likert scale items^*^	Q1: Did all the team members participate in answering the questions given to them?	4.31 ± 0.71	4.5	5
	Q2: Did you feel that you can express your opinion easily in your team?	4.27 ± 0.72	4	5
	Q3: The completion of the preparation material aided my understanding in the lecture?	4.23 ± 0.76	4	5
	Q4: The doctor discussed team-based learning confidently	4.41 ± 0.76	5	5
	Q5: I think the number of group members (10) enhanced my learning process and my peer learning?	4.05 ± 0.86	4	5
	Q6: The doctor helped to focus discussions and learning?	4.35 ± 0.79	5	5
	Q7: I received useful and timely feedback from my tutor	4.31 ± 0.73	5	5
	Q8: I enjoyed team-based learning more than lecture-based learning	4.08 ± 0.9	4	5
	Q9: I believe team-based learning motivated my participation in the learning process	4.16 ± 0.8	4	5
	Q10: The team discussed multiple points of views while answering the given questions	4.32 ± 0.74	5	5
	Q11: The individual and team assessments (iRAT and tRAT) aided my understanding	4.12 ± 0.75	4	4

^*^1: Strongly disagree, 2: Disagree, 3: Neutral, 4: Agree, 5: Totally agree Data are presented as mean ± standard deviation

**Fig. 3:**
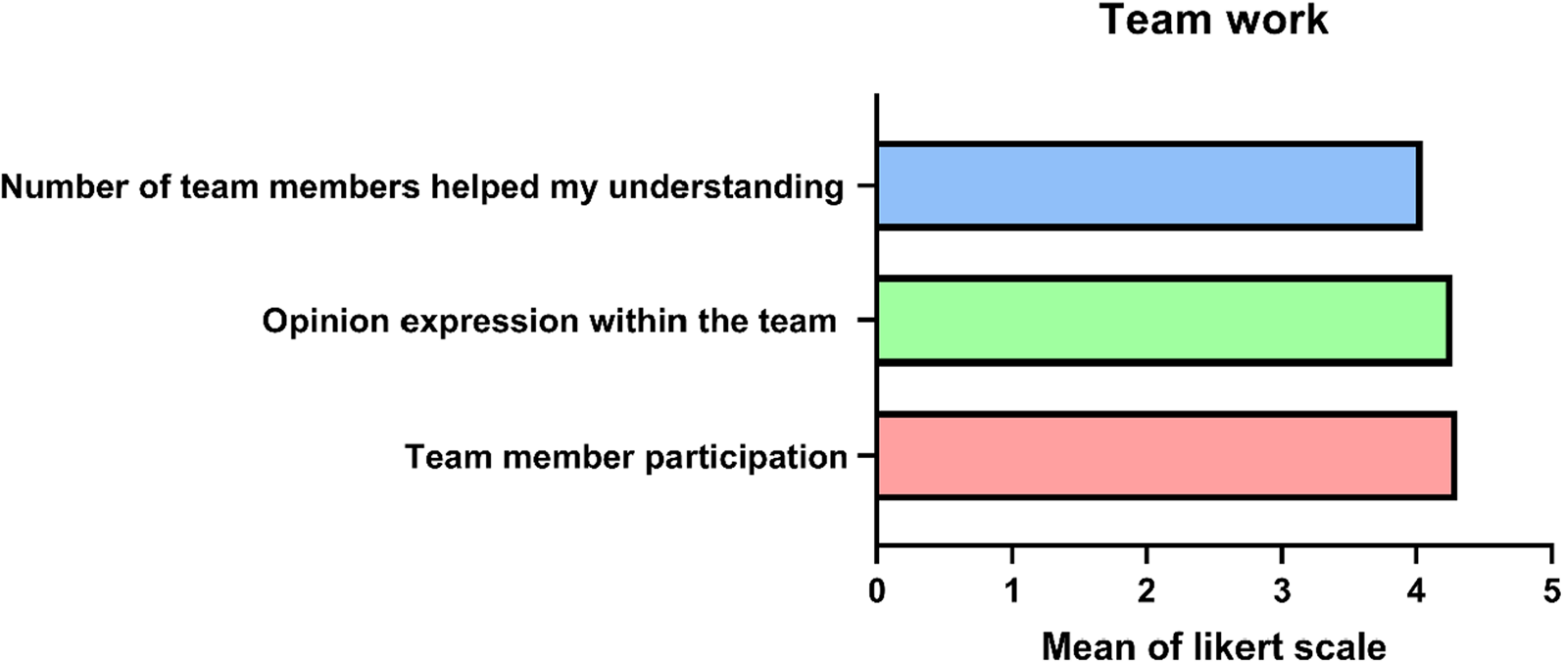
The students’ opinions about the learning process in teams. Data are presented as mean ± SD. *n* = 116, where 1 is the least and 5 is the most

Moreover, the opinions of the learners were collected regarding the efficiency of team structure and their participation during the TBL session; 78.5% of the students strongly agreed/agreed that number of team members (*n* = 10) helped their understanding of the topic through active discussions. Moreover, 93.5% of the students strongly agreed/agreed that they could express themselves easily in their teams and didn’t have any problem concerning participation. Furthermore, 90.5% strongly agreed/agreed that all the team members participated in answering the questions of the tRat and they voted on the correct answer. Regarding the factors that affect the TBL sessions, 99% of the students confirmed that the completion and the quality of the provided preparation materials aided them in their understanding (Fig. 3, Table 1).

**Fig. 4:**
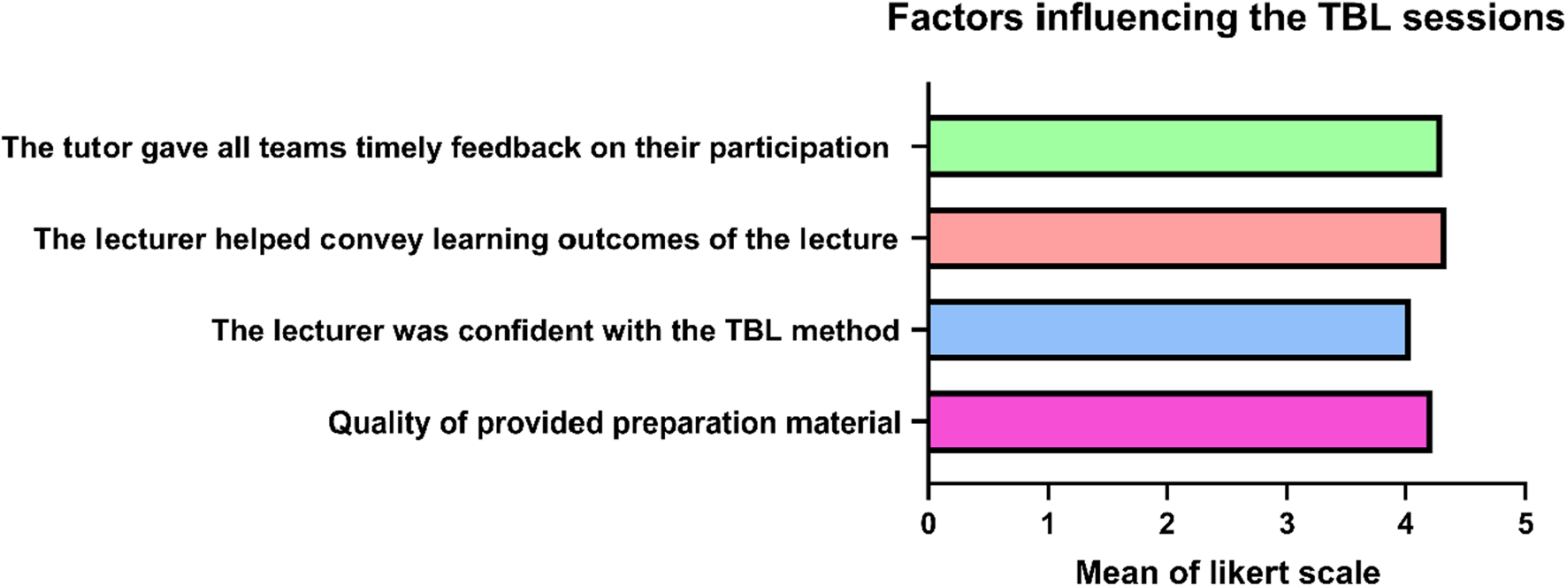
Different factors influencing the TBL sessions. Data are presented as mean ± SD. *n* = 116. TBL: Team-based learning, where 1 is the least and 5 is the most

Around 85% of the students thought that their lecturers discussed and lead the TBL session confidently, in addition to being able to focus, discuss and convey important learning outcomes to them easily and that they received timely feedback on their questions and participation during the session (Fig. 4, Table 1).

**Fig. 5:**
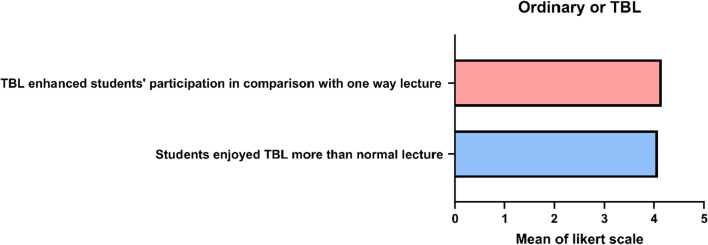
The students’ opinions regarding comparing the normal lecture with team-based learning. Data are presented as mean ± SD. *n* = 116. TBL: team-based learning, where 1 is the least and 5 is the most

**Fig. 6:**
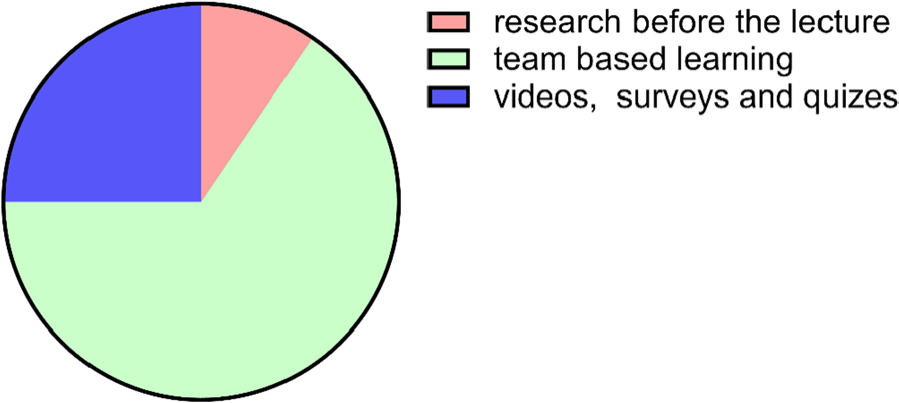
Pie chart showing the students’ suggestions to lead the way to more engaging lectures

Actually, the students’ gratitude and satisfaction towards the TBL lead lecture were very high compared with the one-way conventional lecture as 80% of the students stated that they enjoyed the TBL session and 85% of them stated that the TBL method motivated their participation and enhanced their learning experience (Fig. 5, Table 1). Finally, an open-ended question was assessing the students’ other suggestions to maximise the educational process; to our amazement, 65.5% of the students stated that they recommend the application of TBL in all the courses as it increased their enjoyment and their benefit from this method of learning (Fig. 6, Table 2).

**Table 2:** Students’ suggestions for more engaging lecture

Type of question	content	Students’ suggestions	Overall percentage
Open ended question	Suggest another method of teaching to get the most benefit out of the educational process	By encouraging students to conduct topic research before the lecture	9.5
		By using the team-based learning method	65.5
		By using more in lecture animated videos, surveys and quizzes	25

The Cronbach’s α of the collected responses were 1.04 showing excellent internal consistency of the questions. The students who answered this survey were 66.4% females, their ages were ranged between 19 and 22 years old.

## Discussion

The team-based learning is one of the teaching methods that were proven to increase the participation of the students and that overcomes the one-way lecture [1, 2]. It is one of the prominent active-learning strategies that were applied in healthcare education over the last few decades [5]. The cooperative nature of TBL inspires medical students to develop their communication and partnership skills, providing a beneficial learning experience. First, the topics were designed to cover all the stages of the team-based learning pedagogy; the pre-class materials were supplied for preparation pre-class. Second the readiness assurance tests were distributed (iRAT and tRAT); the TRAT included a case study addressing the lecture’s topic which encouraged the problem solving skills and communication skills of the students. Finally, an immediate feedback was given to the students discussing both distributed tests. Applying the latter was found to increase the student’s engagement during their pharmacology class as well as their medicinal chemistry one.

The survey conducted at the end of the team-based learning sessions measured the student’s gratification, comprehending and overall opinion about this tutoring method. From the survey it was noticed that the student’s overall satisfaction, understanding and participation were fostered compared to other topics that were taught in the ordinary methods. The acceptance of the students was highlighted when they were asked to suggest another method to increase their engagement and comprehending in the lecture; above 50% of the students recommended the TBL method as an appealing method for better perception and participation. This finding goes with the observation highlighted previously that using TBL greatly increase medical students reasoning and understanding [5].

The majority of the participants found it easy to express their opinions within their teams. Moreover, most of the students ensured that they enjoyed the team-based sessions more than the ordinary lecture and thought it motivated them for more participation; this finding assures the theory that was postulated by Abraham Flexener around a decade ago that medical students need to be taught in an active way rather than the passive lectures [11]. Furthermore, most of the students felt that the distributed tests aided their understanding, as mentioned previously by Chad [12] the spread-out tests encouraged them to exert more effort in studying to achieve higher marks. More than half of the participants stated that preparing the reading materials and the videos helped them understand more the topic to be taught, which go well with the finding of Tan et al. [13] that using TBL improved the students’ knowledge to a great extent. Finally, the team-based learning lectures showed excellency as a promising method that helped the students concentrate and participate during the lecture.

## Conclusion

Finally, the study demonstrates that team-based learning could be a valuable way of tutoring Pharmacology and Medicinal Chemistry to undergraduate pharmacy students. Moreover, employing the TBL method was thoroughly understood by the students. In addition, it nurtured the students’ curiosity to learn and convey their thoughts out-loud in the lectures in the premises of the Faculty of Pharmacy in the Future University in Egypt.

## Data Availability

The datasets used and/or analysed during the current study are available from the corresponding author upon reasonable request.

## Abbreviations

TBL: Team-based learning
RAT: Readiness assurance tests
iRAT: Individual readiness assurance tests
tRAT: Team readiness assurance tests
